# Apolipoprotein B and renal function: across-sectional study from the China health and nutrition survey

**DOI:** 10.1186/s12944-020-01241-7

**Published:** 2020-05-27

**Authors:** Wenbo Zhao, Junqing Li, Xiaohao Zhang, Xiaomei Zhou, Junyi Xu, Xun Liu, Zifeng Liu

**Affiliations:** 1 Department of Nephrology, The Third Affiliated Hospital of Sun Yat-sen University, Guangzhou, Tianhe Road NO.600, Guangzhou, China; 2 Department of Clinical Data Center, The Third Affiliated Hospital of Sun Yat-sen University, Guangzhou, China

**Keywords:** Chronic kidney disease, Apolipoprotein B, Risk factor, Dyslipidemia, Atherosclerosis

## Abstract

**Background:**

Chronic kidney disease (CKD) is a worldwide public health problem characterized by changes in kidney structure and function, usually leading to a loss of kidney function. The identification of risk factors and management of patients with early-stage CKD may slow or prevent the progression to end-stage renal disease.

**Methods:**

This study used the population-based cohort database from the China Health and Nutrition Survey (CHNS). Data from 11,978 patients were collected from the 2009 to 2011 wave of the CHNS. After removing patients with missing data, we finally included 8322 participants. A cross-sectional design was used to assess the association between Apolipoprotein B (Apo-B) levels and CKD. We used overlapping covariates to develop 5 models to evaluate the odds ratios.

**Results:**

Among the study participants, patients with estimated glomerular filtration rates (eGFR) < 60 ml/min/1.73m^2^were more likely to have increased Apo-B levels (> 1.2 mmol/L, 19.41%), likely to be elderly (> 65 years, 61.76%), likely to be female (61.21%), and likely to be less educated (< 6 years and > 6 & ≤12 years, 32.07 and 52.44%, respectively).The significant association between Apo-B and CKD defined by eGFR even after adjusting for confounders including demographic characteristics, nutritional status, comorbidities, biochemical indicators, and lifestyle factors. In addition, stratified analyses showed that young and middle age (< 65 years), being overweight (body mass index [BMI] > 25 kg/m^2^), and hyperuricemia were associated with higher risks of CKD stages.

**Conclusions:**

The results of this Chinese population-based study revealed a strong positive correlation between Apo-B and CKD stages. The current findings were obtained from an epidemiologic study; therefore, these data cannot directly address the mechanisms of disease progression. The underlying mechanisms require analysis in future independent validation and prospective cohort studies.

## Background

Chronic kidney disease (CKD) is a worldwide public health problem characterized by changes in kidney structure and function usually leading to a loss of kidney function [1]. A national survey reported a CKD prevalence of 10.8% (approximately 120 million patients) among the adult Chinese population [2].The identification of risk factors and management of patients with early-stage CKD may slow or prevent the progression to end-stage renal disease (ESRD).

Dyslipidemia is common among patients with CKD [3]. Serum lipid levels are linked to atherosclerotic diseases and lipid and lipoprotein ratios are risk factors for atherosclerosis with renal failure [4]. ESRD is associated with accelerated atherosclerosis and a high incidence of cardiovascular disease. Higher very-low-density lipoprotein cholesterol (VLDL-C) and apolipoprotein B (Apo-B) levels and lower high-density lipoprotein cholesterol (HDL-C) and Apo-A1 levels are associated with an increased risk for arteriosclerotic cardiovascular disease (ASCVD) [5]. Dyslipidemia is associated with a reduction of the glomerular filtration rate (GFR). Apo-B levels are increased in CKD stages 1–5 [6]. CKD also delays the catabolism of VLDL-Apo-B particles [7]. The accumulation of Apo-B-containing lipoproteins may result from decreased lipoprotein clearance rather than from increased synthesis [8]. Animal studies have shown the development and progression of kidney damage in the setting of hyperlipidemia with increased glomerulosclerosis and tubule interstitial damage [9, 10]. Thus, serum lipids may be independent risk factors for CKD stages. In addition, our small sample study found that Apo-B was associated with the progression of diabetic kidney disease [11], so we put forward a hypothesis that Apo-B may be associated with CKD levels.

Although the association between Apo-B and CKD stages has been evaluated [12, 13], the results of previous studies are inconsistent and the relationship between Apo-B levels and changes in renal function is not clear. In addition, no studies have included large samples of Chinese cohorts. This large cross-sectional study aimed to analyze the relationship between Apo-B levels and the stages of CKD in participants of the China Health and Nutrition Survey (CHNS).

## Methods

### Data resource

**Fig. 1:**
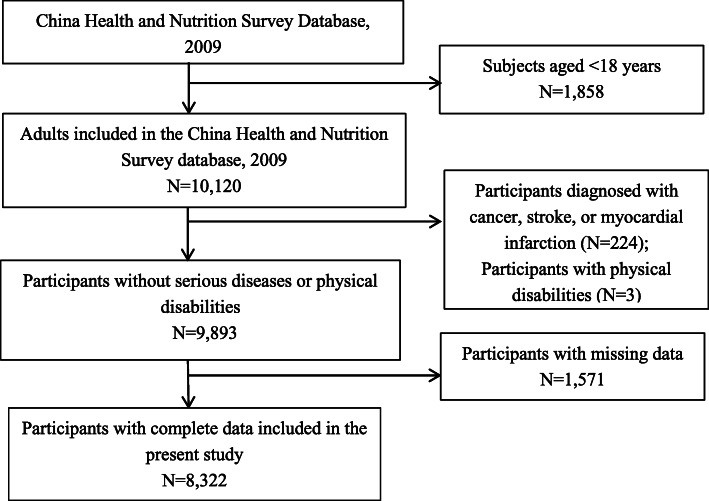
Study flowchart

This study used data from the population-based cohort database from China Health and Nutrition Survey (CHNS). The CHNS database included data for more than 15,000 individuals in approximately nine provinces from 1989 to2011.All participants provided written informed consents. The survey collected comprehensive demographic data including sex, age, education, income level, diet and nutritional status, health status and use of health services, lifestyle data, and limited clinical data [14]. We identified 11,978 participants from the 2009 CHNS wave. We defined the subjects of this study as patients > 18 years of age and those without serious diseases or physical disabilities. Serious diseases or physical disability may result in malnutrition, which may be a confounder and affect the determination of serum creatinine and lead to inaccurate estimation of renal function. After removing participants with missing data, the analyses included data from 8322 participants (Fig. 1).

### Exposure definition

Fasting serum was collected and detected Apo-B concentration by immunoturbidimetry. In this study, Apo-B measurements were defined as exposure. We collected data on the participants’ levels of blood biochemical indicators at the time of the 2009 survey and divided the Apo-B levels into 3 groups (≤95, 95–120, and > 120 mg/dL) as described previously [15].

### definition

We determined the stage of CKD according to the calculated glomerular filtration rate (GFR) index. The eGFR was classification into 5 levels according to the K/DOQI and KDIGO Clinical Practice Guidelines for Chronic kidney Disease [16], as follows: 90 ml/min/1.73m^2^ (level 1), > 60 ml/min/1.73m^2−^and 90 ml/min/1.73m^2^ (level 2), > 45 ml/min/1.73m^2^ and ≤ 60 ml/min/1.73m^2^ (level 3), > 45 ml/min/1.73m^2^ and ≤ 30 ml/min/1.73m^2^ (level 4), and ≤ 30 ml/min/1.73m^2^ (level 5). The Chronic Kidney Disease Epidemiology Collaboration equation [17] was used to calculate estimated GFR (eGFR) in milliliters per minute per 1.73 m^2^.

### Covariate selection

Age, sex, body mass index (BMI), region, education level, nutritional status, comorbidities (e.g., hyperuricemia, diabetes, and hypertension), biochemical indicators (e.g., triglycerides, total cholesterol, HDL-C, and low-density lipoprotein) and lifestyle factors (e.g., smoking, alcohol, and sleeping) are all possible confounders of the association between Apo-B and GFR. These covariates were selected based on previous studies [18, 19]. We divide the subjects education into 5 levels according to the number of years completed (6 years in primary school, 3 years in middle school, 3 years in high school, 4–5 years in college, and 2–3 years). Sleep duration was divided into 3levels according to World Health Organization (WHO) guidelines for adults. The biochemical indices (including triglycerides, total cholesterol, HDL-C, and low-density lipoprotein) were categorized as described previously [14].

### Statistical analysis

The present study used a cross-sectional design to assess the association between Apo-B levels and CKD. We conducted ordered multiple logistic regressions to estimate 5 multivariable models adjusting for age, sex, BMI, region, education level, nutritional status, comorbidities, biochemical indicators, and lifestyle factors. We used overlapping covariates to generate 5 models and evaluated the odds ratios (ORs). Model 1 included no covariates, Model 2 was adjusted for demographic characteristics, Model 3 was additionally adjusted for nutritional status factors, Model 4 was additionally adjusted for biochemical indicators, and Model 5 adjusted for all covariates. Secondary analysis estimated the ORs between Apo-B and CKD levels among age groups, sex, BMI, and hyperuricemia groups, in each of the 5models. All analyses were conducted using R (version 3.5.1), and *P*-values < 0.05 were considered statistically significant. The Akaike Information Criterion (AIC) was used to screen models.

### Sensitivity analysis

To validate our conclusions and find possible biases, we further performed a sensitivity analysis. In contrast to our main analysis, we performed correlation analysis on eGFR and Apo-B without encoding them to categorical variables. The results of the sensitivity analysis were compared to those of main analysis to assess whether our findings would be robust.

## Results

A total of 8322 participants (3878 men and 4444 women) aged 18–98 years were included in this study. Their estimated (eGFRs) were categorized into 5 levels. 11.09% of 8322 participants (923) had an eGFR of < 60 ml/min/1.73m^2^, defined as chronic kidney disease (CKD). The basic demographic and clinical characteristics of the study participants are presented in Table 1.

**Table 1:** Demographic and clinical characteristics of subjects included in study

Categorical variable	Total	Level 1^b^		Level 2^b^		Level 3^b^		Level 4^b^		Level 5^b^	
		N (ratio)									
**All**	8322	2233	(0.27)	5166	(0.62)	754	(0.09)	139	(0.02)	30	(0.00)
**APO_B**^a^**, mg/dL**											
95	5085 (61.10)	1692	(75.77)	2954	(57.18)	361	(47.88)	62	(44.60)	16	(53.33)
95 & < 120	2150 (25.84)	394	(17.65)	1483	(28.71)	227	(30.12)	41	(29.50)	5	(16.67)
> 120	1087 (13.06)	147	(6.58)	729	(14.11)	166	(22.00)	36	(25.90)	9	(30.00)
**Age Groups, years**											
18–40	2298 (27.61)	1318	(59.03)	970	(18.78)	8	(1.06)	2	(1.44)	0	(0.00)
40–65	4682 (56.26)	883	(39.54)	3456	(66.90)	305	(40.45)	30	(21.58)	8	(26.67)
> 65	1342 (16.13)	32	(1.43)	740	(14.32)	441	(58.49)	107	(76.98)	22	(73.33)
**Gender**											
Female	4444 (53.40)	1001	(44.83)	2878	(55.71)	456	(60.48)	90	(64.75)	19	(63.33)
Male	3878 (45.60)	1232	(55.17)	2288	(44.29)	298	(39.52)	49	(35.25)	11	(36.67)
**Education, years**											
< =6	2187 (26.27)	482	(21.59)	1409	(27.27)	250	(33.16)	35	(25.18)	11	(36.67)
> 6 & < =12	4139 (49.74)	1115	(49.93)	2540	(49.17)	381	(50.53)	89	(64.03)	14	(46.67)
> 12 & < =16	1581 (18.90)	495	(22.17)	972	(18.82)	100	(13.26)	10	(7.19)	4	(13.33)
> 16	415 (4.99)	141	(6.31)	245	(4.74)	23	(3.05)	5	(3.60)	1	(3.33)
**Body Mass Index, kg/m**^**2**^											
< =25	5906 (70.97)	1675	(75.01)	3584	(69.48)	530	(70.29)	94	(67.63)	23	(97.67)
> 25	2416 (29.03)	558	(24.99)	1582	(30.62)	224	(29.71)	45	(32.37)	7	(2.33)
**Region**											
Rural	5590 (67.17)	1564	(70.01)	3451	(66.80)	471	(62.47)	84	(60.43)	20	(67.67)
Urban	2732 (32.83)	669	(29.99)	1715	(33.20)	283	(37.53)	55	(39.57)	10	(33.33)
**Hyperuricemia**											
No	7042 (84.60)	2031	(90.95)	4408	(85.33)	529	(70.16)	62	(44.60)	12	(40.00)
Yes	1280 (15.40)	202	(9.05)	758	(14.67)	225	(29.84)	77	(55.40)	18	(60.00)
**Diabetes**											
No	7528 (90.46)	2162	(96.82)	4679	(90.57)	577	(76.53)	89	(64.03)	21	(70.00)
Yes	794 (9.54)	71	(3.18)	487	(9.43)	177	(23.47)	50	(35.97)	9	(30.00)
**Hypertension**											
No	7249 (87.11)	1948	(87.24)	4484	(86.80)	669	(88.73)	119	(85.61)	29	(96.67)
Yes	1073 (12.89)	285	(12.76)	682	(13.20)	85	(11.27)	20	(14.39)	1	(3.33)
**Anemia**											
Yes	508 (13.67)	126	(5.64)	272	(5.27)	78	(10.34)	14	(6.72)	18	(60.00)
No	7814 (86.33)	2107	(94.36)	4894	(94.73)	676	(89.66)	125	(93.28)	12	(40.00)
**Smoking**											
No	5761 (69.23)	1474	(66.01)	3603	(52.50)	548	(72.68)	111	(79.86)	25	(83.33)
Yes	2561 (30.77)	759	(33.99)	1563	(47.50)	206	(27.32)	28	(20.14)	5	(16.67)
**Alcohol Drinking**											
No	5621 (67.54)	1363	(61.04)	3520	(68.14)	586	(77.72)	122	(87.77)	30	(1.00)
Yes	2701 (32.46)	870	(38.96)	1646	(31.86)	168	(22.28)	17	(12.23)	0	(0.00)
**Tea Drinking**											
No	5399 (64.88)	1555	(69.64)	3291	(63.70)	449	(59.55)	85	(61.15)	19	(63.33)
Yes	2923 (35.12)	678	(30.36)	1875	(36.30)	305	(40.45)	54	(38.85)	11	(36.67)
**Sleeping Duration, hours/ day**											
< =7	2494 (29.97)	519	(23.24)	1660	(32.13)	263	(34.88)	45	(32.37)	7	(23.33)
> 7 & < =9	4910 (59.00)	1436	(64.31)	3026	(58.58)	368	(48.81)	64	(46.04)	16	(53.33)
> 9	918 (11.03)	278	(12.45)	480	(9.29)	123	(16.31)	30	(21.58)	7	(23.33)
**Albumin, g/dL**											
< =35	44 (0.53)	12	(0.54)	17	(0.33)	9	(1.19)	4	(2.88)	2	(6.67)
> 35 & < =51	7948 (95.51)	2068	(92.61)	4991	(96.61)	731	(96.95)	131	(94.24)	27	(90.00)
> 51	330 (3.97)	153	(6.85)	158	(3.06)	14	(1.86)	4	(2.88)	1	(3.33)
**Triglycerides**											
Ideal	5700 (68.49)	1608	(72.01)	3496	(67.67)	488	(64.72)	84	(60.43)	24	(80.00)
Borderline high	939 (11.28)	207	(9.27)	606	(11.73)	97	(12.86)	28	(20.14)	1	(3.33)
High	1495 (17.96)	350	(15.67)	954	(18.47)	161	(21.35)	25	(17.99)	5	(16.67)
Very high	188 (2.26)	68	(3.05)	110	(2.13)	8	(1.06)	2	(1.44)	0	(0.00)
**Total Cholesterol**											
Desirable	5564 (66.86)	1766	(79.09)	3305	(63.98)	399	(52.92)	76	(54.68)	18	(60.00)
Borderline high	1976 (23.74)	337	(15.09)	1378	(26.67)	223	(29.58)	30	(21.58)	8	(26.67)
High	782 (9.40)	130	(5.82)	483	(9.35)	132	(17.51)	33	(23.74)	4	(13.33)
**HDL**^a^											
Low	2095 (25.17)	534	(23.91)	1317	(25.49)	198	(26.26)	36	(25.90)	10	(33.33)
Normal	3533 (42.45)	992	(44.42)	2195	(42.49)	297	(39.39)	55	(39.57)	12	(40.00)
High	2694 (32.37)	707	(31.66)	1654	(32.02)	277	(36.74)	48	(34.53)	8	(26.67)
**LDL**^a^											
Optimal	2992 (35.95)	1132	(50.69)	1642	(31.78)	181	(24.01)	28	(20.14)	9	(30.00)
Near optimal	2752 (33.07)	685	(30.68)	1771	(34.28)	239	(31.70)	50	(35.97)	7	(23.34)
Borderline high	1699 (20.42)	300	(13.43)	1170	(22.65)	189	(25.07)	30	(21.58)	10	(33.33)
High	624 (7.50)	79	(3.54)	425	(8.23)	101	(13.40)	18	(12.95)	1	(3.33)
Very high	255 (3.06)	37	(0.15)	158	(3.06)	44	(5.84)	13	(9.35)	3	(10.00)
**Urea Nitrogen, mg/dL**											
< =7.1	7019 (84.34)	2032	(91.00)	4370	(84.59)	554	(73.47)	60	(43.17)	3	(10.00)
> 7.1	1303 (15.66)	201	(9.00)	796	(15.41)	200	(26.53)	79	(56.83)	27	(90.00)
**Continuous variable**	Mean (SD)										
**Screen Time, hours/day**	2.19 (1.66)	2	(1.69)	2	(1.65)	2	(1.60)	2	(1.46)	2	(1.89)
**Water Drinking, cups/day**	3.45 (1.82)	3	(1.81)	3	(1.89)	3	(1.41)	4	(1.73)	3	(1.08)
**Fat Intake, g/day**	74.97 (41.19)	74	(44.28)	76	(40.96)	72	(34.13)	69	(32.02)	73	(37.08)
**Protein Intake, g/day**	66.00 (22.96)	69	(24.04)	66	(22.58)	60	(20.97)	54	(17.64)	54	(21.99)
**Caloric Intake, kcal/day**	885.37 (306.41)	941	(318.17)	881	(298.28)	789	(293.42)	719	(264.65)	731	(292.00)

Level 1: GFR > 90 ml/min/1.73m^2^;

Level 2: 60 ml/min/1.73m^2^ < GFR < =90 ml/min/1.73m^2^;

Level 3: 45 ml/min/1.73m^2^ < GFR < =60 ml/min/1.73m^2^;

Level 4: 45 ml/min/1.73m^2^ < GFR < =30 ml/min/1.73m^2^;

Level 5: < 30 ml/min/1.73m^2^

^a^*APO_B* apo-lipoprotein B, *HDL* high-density lipoprotein, *LDL* low-density lipoprotein

^b^ Classification follow the K/DOQI Clinical Practice Guidelines for Chronic Kidney Disease,

A higher proportion of participants with an eGFR of < 60 ml/min/1.73m^2^ had increased Apo-B levels (> 1.2 mmol/L, 22.86%), were elderly (> 65 years, 61.76%), were female (61.21%), and were less education (≤6 years and > 6 & ≤12 years, 32.07 and 52.44%, respectively).

A higher proportion of patients with levels 3–5 CKD or an eGFR< 60 ml/min/1.73m^2^, Apo-B levels > 1.2 mmol/L, were aged > 65 years, were females, were educated (< 6 years and > 6 & ≤ 12 years), had hyperuricemia, and diabetes, slept for (> 7 and ≤ 9 h a day), had high total cholesterol, and high LDL-C levels, drank tea and had urea nitrogen levels > 7.1 mg/dL and had a lower eGFR than the group overall.

We conducted ordered multiple logistic regressions to estimate 5 multivariable models adjusting for age, sex, BMI, region, education level, nutritional status, comorbidities, biochemical indicators, and lifestyle factors. The ORs used to describe the association between Apo-B and CKD were 1.78, 1.48, 1.39,1.28, and 1.29, respectively, for these models. All ORs were statistically significant (Table 2).

**Table 2:** Association of Chronic Kidney Disease and Apo-lipoprotein B in the study subjects *(N =* 8322*)*

Variable	Model 1^c^	Model 2^c^	Model 3^c^	Model 4^c^	Model 5^c^
**OR**^a^	1.78	1.48	1.39	1.28	1.29
**(95% CI**^a^**)**	(1.67, 1.89)	(1.38, 1.58)	(1.29, 1.49)	(1.14, 1.44)	(1.15, 1.45)
**LR**^b^	− 7784.8	− 6494.3	− 6307.2	− 6193.2	− 6156.3
**Chi-Square (DF)**	/	2581.00 (5)	374.30 (7)	227.90 (6)	73.83 (6)
**t value**	17.81	11.33	9.35	4.16	4.25
**p (Apo B)**	/	< 0.01	< 0.01	< 0.01	< 0.01

^a^*OR* Odds ratio, *CI* Confidence interval

^b^ LR test wasperformed between model 1~model 2, model 2~model 3, model 3~model 4, and model4~model5; All the *P* value of LR is < 0.001

^c^Model 1: Estimate without covariate

Model 2: Adjusted for demographic characteristics (age, gender, body mass index, region and education)

Model 3: Adjusted for model 2, nutrient status (fat intakes, protein intakes and calorie intakes) and comorbidities (Hyperuricemia, diabetes, anemia and hypertension)

Model 4: Adjusted for model 3 and biochemical indicators (hemoglobin, albumin, apo-lipoprotein A, urea nitrogen, triglycerides, total cholesterol, high-density lipoprotein and low-density lipoprotein)

Model 5: Adjusted for model 4 and life style (smoking, alcohol drinking, water drinking, tea drinking, screen view and sleeping duration)

**Fig. 2:**
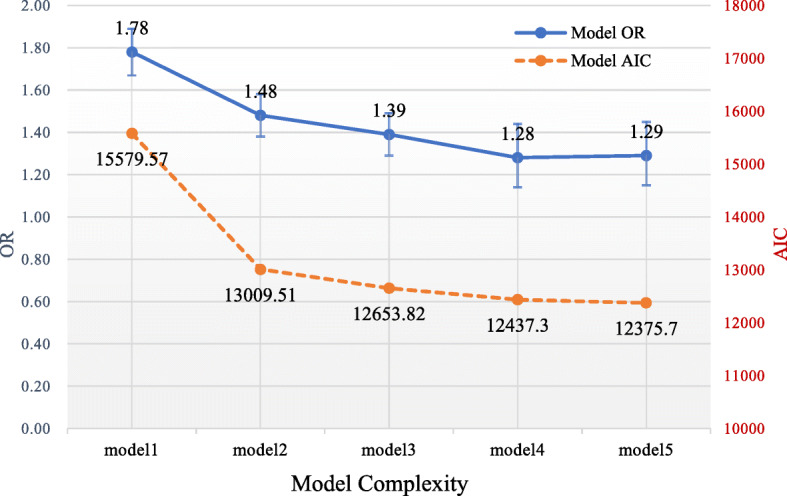
Trend of OR and AIC according to the variation of model complexity

We calculated the Akaike Information Criterion (AIC) for the 5 models used in this study. As shown in Fig. 2, the AIC values of the 5 models gradually declined and leveled off. Model 4 had a smaller AIC (12,437.3) but included the least number of covariate variables, indicating its superior goodness of fit (GoF). Thus, we used Model 4 for observation (OR = 1.28).

The associations between the risk of CKD and increased Apo-B levels using 5 models, stratified by age, sex, BMI and hyperuricemia, are shown in Table 3. In Model 4, the 18–40-years group (OR: 1.41, 95% confidence interval [CI]: 1.11–1.79), 40–65-years age group (OR: 1.30, 95%CI: 1.11–1.53), male sex (OR: 1.41, 95%CI: 1.20–1.66), BMI > 25 kg/m^2^(OR: 1.60, 95%CI: 1.31–1.96), and hyperuricemia (OR: 1.50, 95%CI: 1.16–1.93) showed significantly increased associations. The remaining factors showed non-significant associations with CKD.

**Table 3:** Association of chronic kidney disease and apo-lipoprotein B in study subjects stratified by age, gender, body mass index and hyperuricemia

Characters	Odds Ratio (95% CI^a^)				
	Model 1^b^	Model 2^b^	Model 3^b^	Model 4^b^	Model 5^b^
**Age Groups**					
18–40	1.53 (1.33,1.77)	1.59 (1.37,1.84)	1.50 (1.29,1.75)	1.41 (1.11,1.79)	1.37 (1.08,1.7)
40–65	1.57 (1.44,1.73)	1.59 (1.37,1.84)	1.51 (1.37,1.66)	1.30 (1.11,1.53)	1.32 (1.12,1.55)
> 65	1.28 (1.12,1.47)	1.19 (1.03,1.37)	1.14 (0.99,1.33)	1.14 (0.87,1.49)	1.18 (0.90,1.55)
**Gender**					
Female	2.03 (1.86,2.22)	1.45 (1.32,1.60)	1.36 (1.23,1.50)	1.13 (0.96,1.35)	1.14 (0.96,1.36)
Male	1.55 (1.42,1.70)	1.49 (1.35,1.65)	1.42 (1.28,1.57)	1.41 (1.20,1.66)	1.41 (1.20,1.67)
**Body Mass Index**					
< =25	1.79 (1.66,1.94)	1.48 (1.37,1.61)	1.37 (1.25,1.49)	1.15 (0.99,1.34)	1.16 (0.99,1.34)
> 25	1.74 (1.55,1.94)	1.51 (1.35,1.70)	1.45 (1.28,1.63)	1.60 (1.31,1.96)	1.62 (1.32,1.99)
**Hyperuricemia**					
Yes	1.43 (1.25,1.64)	1.38 (1.19,1.59)	1.40 (1.21,1.63)	1.50 (1.16,1.93)	1.52 (1.18,1.97)
No	1.75 (1.63,1.88)	1.42 (1.31,1.45)	1.39 (1.29,1.51)	1.19 (1.04,1.36)	1.19 (1.04,1.37)

^a^*CI* Confidence interval

^b^Adjusted for the same model as Table 2 except the variable that are stratified

### Sensitivity Analysis

The correlation analysis of Apo-B and eGFR as the quantitative variables, indicated negative correlation between them(*r* = − 0.26, *p* < 0.001), which is consistent with the result according to ranges and categories.

## Discussion

The present study demonstrated a correlation between increased Apo-B levels and renal function decline based on across-sectional study of 8322 participants of the CHNS. The significant association between Apo-B and CKD defined by eGFR persisted even after adjusting for confounders including demographic characteristics, nutritional status, comorbidities, biochemical indicators, and lifestyle factors. Model 4 had a smaller AIC (12,437.3) and the least number of covariate variables, meaning superior goodness of fit (GoF). In addition, stratified analyses showed that low age (18–40 years) and middle age (41–65 years), being overweight (BMI > 25 kg/m^2^), and hyperuricemia were associated with CKD levels.

The exact role of apolipoproteins in CKD remains a matter of debate. Although numerous studies have evaluated the association between apolipoproteins and CKD [20–23], few have focused on Chinese populations, particularly large population-based cohorts. In the Chronic Renal Insufficiency Cohort (CRIC) study, Apo-B level was not independently associated with the progression of kidney disease [23]. In another study, Apo-B/A1 level but not Apo-B level was associated with the progression of CKD; however, Apo-B was not [21]. A study on the outcomes of immunoglobulin A (IgA) nephropathy reported the same finding [22]. A large healthy cohort study observed no longitudinal association between incident CKD and baseline Apo-B or the Apo-B/Apo-A1 ratio [20]. However, the present Chinese population-based study revealed a strong positive correlation between Apo-B and CKD stages.

This seemingly paradoxical relationship between Apo-B and CKD remains are unclear. In addition, Apo-B is a predictor of sclerotic cardiovascular disease (ASCVD) and is increasingly recognized as an important risk factor [24, 25]. The association between apolipoproteins and CKD may be partially mediated by the effects of these lipoproteins on genesis. Moreover, the consistent findings before and after adjustment for known common risk factors for sclerosis such as age, diabetes, hypertension and lipoproteins suggested that additional mechanisms beyond atherosclerosis may be involved in the association of apolipoproteins with CKD.

CKD is characterized by specific alterations in lipoprotein metabolism [26]. Hyperlipidemia has been shown to result in glomerular Apo-B accumulation, glomerular hypertrophy, increasing urine albumin, elevating transforming growth factor (TGF-β) levels, and continuous renal injury [27, 28]. An earlier study [29] reported a significant correlation between the plasma concentration of complex, triglyceride-rich Apo-B-containing lipoproteins and the rate of progression but not between cholesterol-rich Apo-B-containing lipoproteins and GFR alterations. Most likely, triglyceride-rich rather than cholesterol-rich lipoprotein particles contribute to the progression of CKD. Complex Apo-B-containing lipoproteins of intermediate and low densities may promote kidney damage through interactions with glomerular and/or tubulointerstitial issues [30].

Apolipoprotein levels differ greatly among different subpopulations. Univariate analyses in the National Health and Nutrition Examination Survey (NHANES) III and Atherosclerosis Risk in Communities (ARIC) studies showed higher mean apolipoprotein A1 and lower apolipoprotein B values in black subpopulations compared to their white counterparts [21]. Combined with the earlier evidence [31], the strong correction between Apo-B level and CKD stages in this study suggests that genetic factors may play an important role in these differences.

Stratified analyse show that patients with hyperuricemia had a higher risk of CKD stages. Consistent with our findings, a comprehensive assessment of the association of dyslipidemia with hyperuricemia in a US adult population reported a linear correlation between Apo-B and LDL cholesterol levels and the ratio of Apo-B to Apo-A1 with serum uric acid levels even after adjusting for covariates including age, sex, and race [32]. Moreover, previous studies have suggested that elevated serum urate levels can contribute to kidney disease, hypertension, and metabolic syndrome [33]. Given the strong correction between hyperuricemia, dyslipidemia, and CKD events, treatment guidelines such as diet (for example Mediterranean-style diet) and lifestyle modifications should be developed to improve CKD care.

This study has several limitations. Firstly, although the relationship remained strong after adjusting for relevant covariates in multivariate analyses, a total of 1571 individuals without necessary data were excluded, which may lead to selection bias if the data missing was not missing at random. At the same time, residual confounding and unmeasured factors may also have contributed to this finding. Secondly, the development of CKD was defined only by eGFR and we did not consider proteinuria as a criterion for defining CKD. This may have resulted in the overall incidence of CKD being underestimated in our study. Also, GFR was estimated using a serum creatinine-based equation rather than a direct measurement, which may have overestimated or under estimated the actual GFR. The CHNS design was rigorous. Our study was reliable to substantiate the findings. Although the data was from 2009, that does not affect the study conclusion.

The results underscore the complicated mechanisms involved in the overall regulation of apolipoproteins, dyslipidemia, atherosclerosis, and CKD. The current findings were obtained from an epidemiologic study; thus, these data cannot directly address the mechanisms of disease progression. The underlying mechanisms await future exploration in independent validation and prospective cohort studies.

## Conclusions

The results of this Chinese population-based study revealed a strong positive correlation between Apo-B and CKD stages. The current findings were obtained from an epidemiologic study; therefore, these data cannot directly address the mechanisms of disease progression. The underlying mechanisms require analysis infuture independent validation and prospective cohort studies.

## Supplementary information

**Additional file 1: Supplement Table 1**. Association of chronic kidney disease and apo-lipoprotein B in young people. **Supplement Table 2.** Association of chronic kidney disease and apo-lipoprotein B in middle-age people. **Supplement Table 3.** Association of chronic kidney disease and apo-lipoprotein B in older people. **Supplement Table 4.** Association of chronic kidney disease and apo-lipoprotein B in female. **Supplement Table 5.** Association of chronic kidney disease and apo-lipoprotein B in male. **Supplement Table 6.** Association of chronic kidney disease and apo-lipoprotein B in people whose BMI is no more than 25. **Supplement Table 7.** Association of chronic kidney disease and apo-lipoprotein B in people whose BMI is higher than 25. **Supplement Table 8.** Association of chronic kidney disease and apo-lipoprotein B in people with hyperuricemia. **Supplement Table 9.** Association of chronic kidney disease and apo-lipoprotein B in people with hyperuricemia.

## Data Availability

Data of CHNS can be viewed and obtained from the following website: https://www.cpc.unc.edu/projects/china

## Abbreviations

CKD: Chronic kidney disease
CHNS: China Health and Nutrition Survey
Apo-B: Apolipoprotein B
eGFR: Estimated glomerular filtration rates
BMI: Body mass index
ESRD: End-stage renal disease
VLDL-C: Very-low-density lipoprotein cholesterol
HDL-C: High-density lipoprotein cholesterol
ASCVD: Arteriosclerotic cardiovascular disease
WHO: World Health Organization
ORs: Odds ratios
AIC: Akaike Information Criterion
GoF: Superior goodness of fit
CRIC: Chronic Renal Insufficiency Cohort
IgA: Immunoglobulin A
TGF-β: Transforming growth factor
NHANES: National Health and Nutrition Examination Survey
ARIC: Atherosclerosis Risk in Communities
