# Supplementary material for: Apolipoprotein B and renal function: across-sectional study from the China health and nutrition survey
Source: Lipids Health Dis. 2020 May 27;19:110. doi: 10.1186/s12944-020-01241-7 (PMC7254739; doi:10.1186/s12944-020-01241-7)
Supplement: Supplementary file 1 — Additional file 1: Supplement Table 1. Association of chronic kidney disease and apo-lipoprotein B in young people. Supplement Table 2. Association of chronic kidney disease and apo-lipoprotein B in middle-age people. Supplement Table 3. Association of chronic kidney disease and apo-lipoprotein B in older people. Supplement Table 4. Association of chronic kidney disease and apo-lipoprotein B in female. Supplement Table 5. Association of chronic kidney disease and apo-lipoprotein B in male. Supplement Table 6. Association of chronic kidney disease and apo-lipoprotein B in people whose BMI is no more than 25. Supplement Table 7. Association of chronic kidney disease and apo-lipoprotein B in people whose BMI is higher than 25. Supplement Table 8. Association of chronic kidney disease and apo-lipoprotein B in people with hyperuricemia. Supplement Table 9. Association of chronic kidney disease and apo-lipoprotein B in people with hyperuricemia. [file 12944_2020_1241_MOESM1_ESM.docx]

| Supplement Table 1: Association of chronic kidney disease and apo-lipoprotein B in young people | | | | | |
| --- | --- | --- | --- | --- | --- |
| Variable ^$^ | Model 1 | Model 2 | Model 3 | Model 4 | Model 5 |
| OR(95% CI*) | 1.53(1.33,1.77) | 159(1.37,1.84) | 1.50 (1.29,1.75) | 1.41 (1.11,1.79) | 1.37 (1.08,1.75) |
| LR* | -1611.00 | -1602.50 | -1575.80 | -1563.30 | -1545.40 |
| Chi-Square(DF) | / | 16.98(4) | 53.37(7) | 25.08(6) | 35.78(6) |
| t-value | 5.91 | 6.15 | 5.30 | 2.78 | 2.56 |
| DF | 2293.00 | 2289.00 | 2282.00 | 2276.00 | 2270.00 |
| p（Apo-lipoprotein B） | <0.01 | <0.01 | <0.01 | <0.01 | <0.01 |
| $ Model 1: Estimate without covariate.  Model 2: Adjusted for demographic characteristics (age, gender, body mass index, region and education).  Model 3: Adjusted for model 2, nutrient status (fat intakes, protein intakes and calorie intakes) and comorbidities (Hyperuricemia, diabetes, anemia and hypertension).  Model 4: Adjusted for model 3 and biochemical indicators (hemoglobin, albumin, apo-lipoprotein A, urea nitrogen, triglycerides, total cholesterol, high-density lipoprotein and low-density lipoprotein). Model 5: Adjusted for model 4 and life style (smoking, alcohol drinking, water drinking, tea drinking, screen view and sleeping duration).  * LR test was performed between model 1 vs. model 2, model 2 vs. model 3, model 3 vs. model 4, and model4 vs. model5;  the P value of LR is < 0.001 | | | | | |

| Supplement Table 2: Association of chronic kidney disease and apo-lipoprotein B in middle-age people | | | | | |
| --- | --- | --- | --- | --- | --- |
| Variable | Model 1 | Model 2 | Model 3 | Model 4 | Model 5 |
| OR(95% CI) | 1.57(1.44,1.73) | 1.59(1.37,1.84) | 1.51(1.37,1.66) | 1.30(1.11,1.53) | 1.32(1.12,1.55) |
| LR^$^ | -3509.10 | -3457.10 | -3370.10 | -3311.80 | -3287.50 |
| Chi-Square | / | 104(4) | 174(7) | 116.57(6) | 48.58(6) |
| t-value(df) | 9.69 | 9.78 | 8.55 | 3.29 | 3.41 |
| DF | 4677.00 | 4673.00 | 4666.00 | 4660.00 | 4654.00 |
| p（Apo-lipoprotein B） | <0.01 | <0.01 | <0.01 | <0.01 | <0.01 |
| $ Model 1: Estimate without covariate.  Model 2: Adjusted for demographic characteristics (age, gender, body mass index, region and education).  Model 3: Adjusted for model 2, nutrient status (fat intakes, protein intakes and calorie intakes) and comorbidities (Hyperuricemia, diabetes, anemia and hypertension).  Model 4: Adjusted for model 3 and biochemical indicators (hemoglobin, albumin, apo-lipoprotein A, urea nitrogen, triglycerides, total cholesterol, high-density lipoprotein and low-density lipoprotein). Model 5: Adjusted for model 4 and life style (smoking, alcohol drinking, water drinking, tea drinking, screen view and sleeping duration).  * LR test was performed between model 1 vs. model 2, model 2 vs. model 3, model 3 vs. model 4, and model4 vs. model5;  the P value of LR is < 0.001 | | | | | |

| Supplement Table 3: Association of chronic kidney disease and apo-lipoprotein B in older people | | | | | |
| --- | --- | --- | --- | --- | --- |
| Variable | Model 1 | Model 2 | Model 3 | Model 4 | Model 5 |
| OR | 1.28(1.12,1.47) | 1.19(1.03,1.37) | 1.14(0.99,1.33) | 1.14(0.87,1.49) | 1.18(0.90,1.55) |
| LR | -1405.70 | -1390.00 | -1308.60 | -1263.30 | -1250.70 |
| Chi-Square | / | 31.22(4) | 162.86 | 90.61 | 25.17 |
| t-value | 3.53 | 2.37 | 1.76 | 0.96 | 1.20 |
| DF | 1337.00 | 1333.00 | 1326.00 | 1320.00 | 1314.00 |
| p（Apo-lipoprotein B） | <0.01 | <0.01 | 0.04 | 0.17 | 0.11 |
| $ Model 1: Estimate without covariate.  Model 2: Adjusted for demographic characteristics (age, gender, body mass index, region and education).  Model 3: Adjusted for model 2, nutrient status (fat intakes, protein intakes and calorie intakes) and comorbidities (Hyperuricemia, diabetes, anemia and hypertension).  Model 4: Adjusted for model 3 and biochemical indicators (hemoglobin, albumin, apo-lipoprotein A, urea nitrogen, triglycerides, total cholesterol, high-density lipoprotein and low-density lipoprotein). Model 5: Adjusted for model 4 and life style (smoking, alcohol drinking, water drinking, tea drinking, screen view and sleeping duration).  * LR test was performed between model 1 vs. model 2, model 2 vs. model 3, model 3 vs. model 4, and model4 vs. model5;  the P value of LR is < 0.001 | | | | | |

| Supplement Table 4: Association of chronic kidney disease and apo-lipoprotein B in female | | | | | |
| --- | --- | --- | --- | --- | --- |
| Variable | Model 1 | Model 2 | Model 3 | Model 4 | Model 5 |
| OR | 2.03(1.86,2.22) | 1.45(1.32,1.60) | 1.36(1.23,1.50) | 1.13(0.96,1.35) | 1.14(0.96,1.36) |
| LR | -4106.6 | -3360 | -3242 | -3162.3 | -3151.7 |
| Chi-Square | / | 1493.2 | 236.01 | 159.4 | 21.2 |
| t-value | 15.71 | 7.51 | 6.07 | 1.44 | 1.54 |
| DF | 4439 | 4435 | 4428 | 4422 | 4416 |
| p（Apo-lipoprotein B） | <0.01 | <0.01 | <0.01 | 0.07 | 0.06 |
| $ Model 1: Estimate without covariate.  Model 2: Adjusted for demographic characteristics (age, gender, body mass index, region and education).  Model 3: Adjusted for model 2, nutrient status (fat intakes, protein intakes and calorie intakes) and comorbidities (Hyperuricemia, diabetes, anemia and hypertension).  Model 4: Adjusted for model 3 and biochemical indicators (hemoglobin, albumin, apo-lipoprotein A, urea nitrogen, triglycerides, total cholesterol, high-density lipoprotein and low-density lipoprotein). Model 5: Adjusted for model 4 and life style (smoking, alcohol drinking, water drinking, tea drinking, screen view and sleeping duration).  * LR test was performed between model 1 vs. model 2, model 2 vs. model 3, model 3 vs. model 4, and model4 vs. model5;  the P value of LR is < 0.001 | | | | | |

| Supplement Table 5: Association of chronic kidney disease and apo-lipoprotein B in male | | | | | |
| --- | --- | --- | --- | --- | --- |
| Variable | Model 1 | Model 2 | Model 3 | Model 4 | Model 5 |
| OR* | 1.55(1.42,1.70) | 1.49(1.35,1.65) | 1.42(1.28,1.57) | 1.41(1.20,1.66) | 1.41(1.20,1.67) |
| LR^$^ | -3617.20 | -3113.30 | -3039.20 | -2991.80 | -2962.50 |
| Chi-Square | / | 1007.60 | 148.36 | 94.70 | 58.60 |
| t-value | 9.50(5) | 8.01(9) | 6.86(16) | 4.08(22) | 4.10(28) |
| DF | 3873.00 | 3869.00 | 3862.00 | 3856.00 | 3850.00 |
| p（Apo-lipoprotein B） | <0.01 | <0.01 | <0.01 | <0.01 | <0.01 |
| $ Model 1: Estimate without covariate.  Model 2: Adjusted for demographic characteristics (age, gender, body mass index, region and education).  Model 3: Adjusted for model 2, nutrient status (fat intakes, protein intakes and calorie intakes) and comorbidities (Hyperuricemia, diabetes, anemia and hypertension).  Model 4: Adjusted for model 3 and biochemical indicators (hemoglobin, albumin, apo-lipoprotein A, urea nitrogen, triglycerides, total cholesterol, high-density lipoprotein and low-density lipoprotein). Model 5: Adjusted for model 4 and life style (smoking, alcohol drinking, water drinking, tea drinking, screen view and sleeping duration).  * LR test was performed between model 1 vs. model 2, model 2 vs. model 3, model 3 vs. model 4, and model4 vs. model5;  the P value of LR is < 0.001 | | | | | |

| Supplement Table 6: Association of chronic kidney disease and apo-lipoprotein B in people whose BMI is no more than 25 | | | | | |
| --- | --- | --- | --- | --- | --- |
| Variable | Model 1 | Model 2 | Model 3 | Model 4 | Model 5 |
| OR* | 1.79(1.66,1.94) | 1.48(1.37,1.61) | 1.37(1.25,1.49) | 1.15(1.00,1.34) | 1.16(1.00,1.34) |
| LR^$^ | -5587.80 | -4629.70 | -4503.70 | -4416.00 | -4385.80 |
| Chi-Square | / | 1916.20 | 252.07 | 175.31 | 60.45 |
| t-value | 14.45(5) | 9.00(9) | 7.06(16) | 1.93(22) | 1.93(28) |
| DF | 5901.00 | 5897.00 | 5890.00 | 5884.00 | 5878.00 |
| p（Apo-lipoprotein B） | <0.01 | <0.01 | <0.01 | 0.03 | 0.03 |
| $ Model 1: Estimate without covariate.  Model 2: Adjusted for demographic characteristics (age, gender, body mass index, region and education).  Model 3: Adjusted for model 2, nutrient status (fat intakes, protein intakes and calorie intakes) and comorbidities (Hyperuricemia, diabetes, anemia and hypertension).  Model 4: Adjusted for model 3 and biochemical indicators (hemoglobin, albumin, apo-lipoprotein A, urea nitrogen, triglycerides, total cholesterol, high-density lipoprotein and low-density lipoprotein). Model 5: Adjusted for model 4 and life style (smoking, alcohol drinking, water drinking, tea drinking, screen view and sleeping duration).  * LR test was performed between model 1 vs. model 2, model 2 vs. model 3, model 3 vs. model 4, and model4 vs. model5;  the P value of LR is < 0.001 | | | | | |

| Supplement Table 7: Association of chronic kidney disease and apo-lipoprotein B in people whose BMI is higher than 25 | | | | | |
| --- | --- | --- | --- | --- | --- |
| Variable | Model 1 | Model 2 | Model 3 | Model 4 | Model 5 |
| OR* | 1.74(1.55,1.94) | 1.51(1.35,1.70) | 1.45(1.28,1.63) | 1.60(1.31,1.96) | 1.62(1.32,1.99) |
| LR^$^ | -2191.40 | -1862.00 | -1796.80 | -1765.90 | -1754.00 |
| Chi-Square | / | 658.73 | 130.50 | 61.74 | 23.88 |
| t-value | 9.73(5) | 6.92(9) | 6.07(16) | 4.57(22) | 4.64(28) |
| DF | 2411.00 | 2407.00 | 2400.00 | 2394.00 | 2388.00 |
| p（Apo-lipoprotein B） | <0.01 | <0.01 | <0.01 | <0.01 | <0.01 |
| $ Model 1: Estimate without covariate.  Model 2: Adjusted for demographic characteristics (age, gender, body mass index, region and education).  Model 3: Adjusted for model 2, nutrient status (fat intakes, protein intakes and calorie intakes) and comorbidities (Hyperuricemia, diabetes, anemia and hypertension).  Model 4: Adjusted for model 3 and biochemical indicators (hemoglobin, albumin, apo-lipoprotein A, urea nitrogen, triglycerides, total cholesterol, high-density lipoprotein and low-density lipoprotein). Model 5: Adjusted for model 4 and life style (smoking, alcohol drinking, water drinking, tea drinking, screen view and sleeping duration).  * LR test was performed between model 1 vs. model 2, model 2 vs. model 3, model 3 vs. model 4, and model4 vs. model5;  the P value of LR is < 0.001 | | | | | |

| Supplement Table 8: Association of chronic kidney disease and apo-lipoprotein B in people with hyperuricemia | | | | | |
| --- | --- | --- | --- | --- | --- |
| Variable | Model 1 | Model 2 | Model 3 | Model 4 | Model 5 |
| OR* | 1.43(1.25,1.64) | 1.38(1.19,1.59) | 1.40(1.21,1.63) | 1.50(1.16,1.93) | 1.52(1.18,1.97) |
| LR^$^ | -1441.20 | -1189.10 | -1170.30 | -1111.90 | -1097.50 |
| Chi-Square | / | 504.10 | 37.60 | 116.83 | 28.78 |
| t-value | 5.14 | 4.34 | 4.51 | 3.10 | 3.21 |
| DF | 7037.00 | 7033.00 | 7026.00 | 7020.00 | 7014.00 |
| p（Apo-lipoprotein B） | <0.01 | <0.01 | <0.01 | <0.01 | <0.01 |
| $ Model 1: Estimate without covariate.  Model 2: Adjusted for demographic characteristics (age, gender, body mass index, region and education).  Model 3: Adjusted for model 2, nutrient status (fat intakes, protein intakes and calorie intakes) and comorbidities (Hyperuricemia, diabetes, anemia and hypertension).  Model 4: Adjusted for model 3 and biochemical indicators (hemoglobin, albumin, apo-lipoprotein A, urea nitrogen, triglycerides, total cholesterol, high-density lipoprotein and low-density lipoprotein). Model 5: Adjusted for model 4 and life style (smoking, alcohol drinking, water drinking, tea drinking, screen view and sleeping duration).  * LR test was performed between model 1 vs. model 2, model 2 vs. model 3, model 3 vs. model 4, and model4 vs. model5;  the P value of LR is < 0.001 | | | | | |

| Supplement Table 9: Association of chronic kidney disease and apo-lipoprotein B in people with hyperuricemia | | | | | |
| --- | --- | --- | --- | --- | --- |
| Variable | Model 1 | Model 2 | Model 3 | Model 4 | Model 5 |
| OR* | 1.75(1.63,1.88) | 1.42(1.31,1.45) | 1.39(1.29,1.51) | 1.19(1.04,1.36) | 1.19(1.04,1.37) |
| LR^$^ | -6209.30 | -5141.80 | -5106.70 | -5042.90 | -5017.20 |
| Chi-Square | / | 2135.00 | 70.14 | 127.66 | 51.37 |
| t-value | 15.21(5) | 8.73(9) | 8.20(16) | 2.54(22) | 2.57(28) |
| DF | 1275.00 | 1271.00 | 1264.00 | 1258.00 | 1252.00 |
| p（Apo-lipoprotein B） | <0.01 | <0.01 | <0.01 | <0.01 | <0.01 |
| $ Model 1: Estimate without covariate.  Model 2: Adjusted for demographic characteristics (age, gender, body mass index, region and education).  Model 3: Adjusted for model 2, nutrient status (fat intakes, protein intakes and calorie intakes) and comorbidities (Hyperuricemia, diabetes, anemia and hypertension).  Model 4: Adjusted for model 3 and biochemical indicators (hemoglobin, albumin, apo-lipoprotein A, urea nitrogen, triglycerides, total cholesterol, high-density lipoprotein and low-density lipoprotein). Model 5: Adjusted for model 4 and life style (smoking, alcohol drinking, water drinking, tea drinking, screen view and sleeping duration).  * LR test was performed between model 1 vs. model 2, model 2 vs. model 3, model 3 vs. model 4, and model4 vs. model5;  the P value of LR is < 0.001 | | | | | |
